# Transformation of Cellulose via Two-Step Carbonization to Conducting Carbonaceous Particles and Their Outstanding Electrorheological Performance

**DOI:** 10.3390/ijms23105477

**Published:** 2022-05-13

**Authors:** Tomas Plachy, Erika Kutalkova, David Skoda, Pavlina Holcapkova

**Affiliations:** Centre of Polymer Systems, University Institute, Tomas Bata University in Zlin, Trida Tomase Bati 5678, 76001 Zlin, Czech Republic; plachy@utb.cz (T.P.); dskoda@utb.cz (D.S.); holcapkova@utb.cz (P.H.)

**Keywords:** cellulose, renewable, carbonization, electrorheology, suspension, conducting

## Abstract

In this study, cellulose was carbonized in two-steps using hydrothermal and thermal carbonization in sequence, leading to a novel carbonaceous material prepared from a renewable source using a sustainable method without any chemicals and, moreover, giving high yields after a treatment at 600 °C in an inert atmosphere. During this treatment, cellulose was transformed to uniform microspheres with increased specific surface area and, more importantly, conductivity increased by about 7 orders of magnitude. The successful transition of cellulose to conducting carbonaceous microspheres was confirmed through SEM, FTIR, X-ray diffraction and Raman spectroscopy. Prepared samples were further used as a dispersed phase in electrorheological fluids, exhibiting outstanding electrorheological effects with yield stress over 100 Pa at an electric field strength 1.5 kV mm^−1^ and a particle concentration of only 5 wt%, significantly overcoming recent state-of-the-art findings. Impedance spectroscopy analysis showed clear interfacial polarization of this ER fluid with high dielectric relaxation strength and short relaxation time, which corresponded to increased conductivity of the particles when compared to pure cellulose. These novel carbonaceous particles prepared from renewable cellulose have further potential to be utilized in many other applications that demand conducting carbonaceous structures with high specific surface area (adsorption, catalyst, filtration, energy storage).

## 1. Introduction

Nowadays, sustainability demands and the emphasis on utilizing renewable material sources are increasing in many applications. Cellulose is the most abundant and renewable natural polymer, and is used in many applications as a fiber material, including wood, construction material, cotton, cellulose particles, etc. It is well known that many organic materials can be carbonized in an inert atmosphere to produce novel carbonaceous conducting structures, further expanding their possible application areas. The carbonization of cellulose, however, commonly gives very low yields, even during its treatment in an inert atmosphere [1], and cellulose is, therefore, mainly carbonized hydrothermally at higher temperatures and pressures [2,3,4,5,6]. Such approach gives, however, rise to non-conducting particles due to the disorder in the structure caused by the transformation of cellulose to polyaromatic hydrochar [5,7]. Since cellulose is an abundant and cheap source, which is often a part of waste material that is not further effectively utilized, it is crucial to introduce a way to transform cellulose particles to conducting carbonaceous structures that could be further widely utilized in many applications instead of conducting polymers, as many hazardous chemicals are used during their synthesis. Contrarily, during carbonization the process, only heat is utilized without any other potential risk.

One of the proposed applications for cellulose particles is their utilization as a dispersed phase in electrorheological (ER) fluids [8,9,10], which are systems whose rheological behavior can be controlled via applied external electric fields [11]. While, in the absence of an electric field, electrically polarizable particles are randomly dispersed in continuous non-conducting medium and the fluid exhibits Newtonian behavior, in its presence, particles align to its direction creating rigid chain-like structures [12]. The fluid then behaves as viscoplastic material with a certain yield stress. This transition from a liquid-like to solid-like state, accompanied with an abrupt change in rheological parameters (viscosity, elastic modulus, yield stress, etc.) is called the ER effect, and can be utilized in many applications to suppress vibration (dampers), to serve as a medium for motion control (haptic devices, brakes, joints), or for controlled lubrication [13].

Since unmodified cellulose-based ER fluids exhibit only slight changes in viscosity, their derivatives, such as cellulose tartrate, carbamate, cellulose phosphate, carboxymethylcellulose, etc., are commonly used. These systems exhibit a moderate ER effect confirmed by a high difference between viscosity in the presence of an electric field and its absence [14,15,16,17,18]. For moderate effects of ER fluids based on pure cellulose, some moisture or adsorbed water on cellulose particles must be present [19,20,21]. Zhang et al. found that the optimal content of water in cellulose particles, regarding values of the yield stress, is around 8.5 wt% [21]. From the application point of view, however, the present moisture is undesirable and can cause the corrosion of the device, or other unpleasant phenomena can occur due to low boiling temperature of water. Therefore, the ER fluids based on cellulose derivatives utilized as an anhydrous dispersed phase are prioritized. Liu et al. combined cellulose and laponite into composite particles leading to values of yield stress of 150.1 Pa at an electric field of strength 2 kV mm^−1^ [22], and modified cellulose with urea for even higher yield stresses of over 270 Pa in the same field and a mass fraction of 15 wt% in anhydrous ER fluids [23]. Cellulose can also be prepared in various forms, such as nanocrystals and nanofibers [13], microfibrillated [24], microcrystalline [25], etc. Recently, an ER fluid based on nanocellulose particles dispersed in castor oil has been used as a biolubricant for the electroactive control of friction behavior [13]. Moreover, as a renewable material, cellulose obtained from spent coffee grounds [26], rice husk-based microcrystalline cellulose [25,27], and kenaf cellulose [17] have been utilized as the dispersed phase in ER fluids.

Besides using derivatives of conventional ER materials to enhance their ER performance, carbonization is another process to develop new carbon-based structures with high specific surface and significantly enhanced electric and dielectric properties. Carbonaceous materials such as carbonaceous nanotubes [28,29], graphene-enriched carbonaceous sheets [30], or carbon nanotubes [31] are commonly used as a dispersed phase in ER fluids due their mild conductivity and excellent dielectric properties giving high ER effects of their silicone–oil fluids. Furthermore, in electrorheology, carbonized particles of polyaniline [32], its oligomers [33], poly (p-phenylendiamine) [34], polyaniline-derived carbonaceous nanotubes [35], and carbonaceous materials prepared by the carbonization of starch/silica precursor [36] have been utilized as a dispersed phase. These particles have exhibited outstanding ER performance and, moreover, can be utilized in many applications implementing conducting or semiconducting materials, or generally as carbon materials with high specific surface area, such as catalysts [37], energy storage material [38], filtration [39], etc.

This study introduces a unique two-step carbonization process that combines hydrothermal carbonization with subsequent carbonization in an inert atmosphere at high temperature to obtain carbonized cellulose. Due to the former treatment, the thermal carbonization gives significantly high yields (>40%) of novel conducting carbonaceous structures from hydrochar-prepared cellulose. Its utilization as a renewable dispersed phase in electrorheology led to outstanding ER effects of their fluids even at low concentration (5 wt%). It should be further mentioned that there are many other applications besides electrorheology demanding conducting and semiconducting particles that could be obtained from green sources by inexpensive and ecological synthesis.

## 2. Results and Discussion

**Figure 1:**
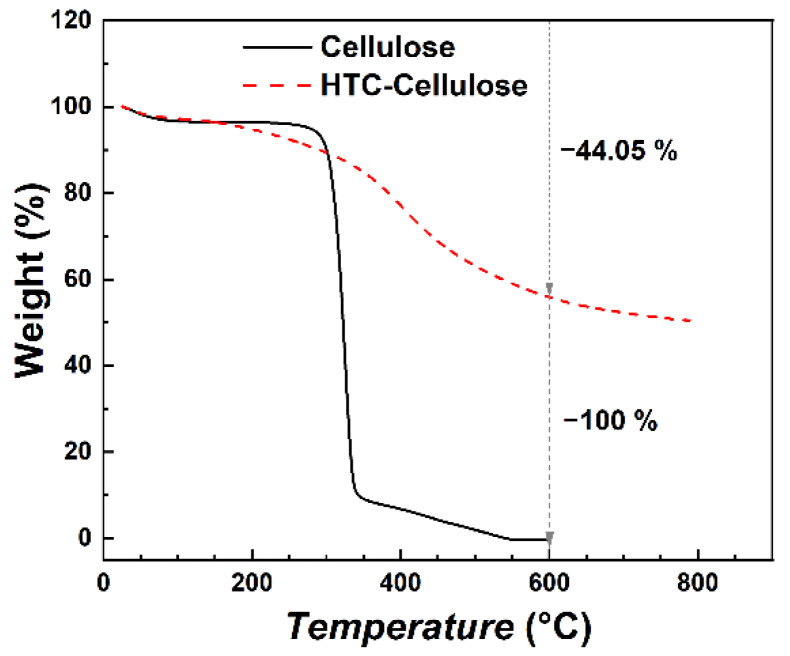
TGA analysis of the cellulose and HTC-Cellulose samples. Measurement ran in a nitrogen atmosphere.

Carbonization is a process commonly used for the transformation of carbon-containing materials into novel materials with enhanced properties. In the case of cellulose, unfortunately, thermal carbonization even in an inert atmosphere gave very low yields of the carbonaceous traces (Figure 1). Before thermal carbonization, therefore, cellulose was hydrothermally carbonized, giving rise to particles further labeled as HTC-Cellulose, which were subsequently thermally carbonized in an argon atmosphere at 600 °C (HTC-TC600-Cellulose), giving a significant yield of novel carbonaceous materials prepared using a green synthesis without any chemicals. For a closer description of the particle preparation process, please see the Section 3. Materials and Methods.

HTC-Cellulose during TGA analysis exhibited a low decrease in weight (~44%), even at 600 °C, thus giving the opportunity to meaningfully use this material as a precursor for the preparation of thermally carbonized materials without the needed addition of any other substances (Figure 1). Since cellulose is one of the most abundant renewable materials, it is desirable to find an approach to transform it to carbonaceous structures with various morphologies, conductivities, etc., that can be further utilized in many applications. It should be mentioned that a yield of the HTC-TC600-Cellulose after the carbonization process in a furnace was slightly lower (~45%) when compared to the TGA analysis, since the sample was held at 600 °C for one hour. Thus, according to mass balance, the hydrothermal carbonization gave a yield of 34.35% of HTC-Cellulose from cellulose. After thermal carbonization, 44.7% of HTC-Cellulose was transformed to HTC-TC600-Cellulose. Overall, approximately 15.35% of the original cellulose was successfully transformed to HTC-TC600-Cellulose using above-mentioned two-step carbonization process. Further optimization of the hydrothermal conditions could lead to significantly higher yields of both HTC-Cellulose and HTC-TC600-Cellulose [6].

While, in the case of pure cellulose, it is hard to obtain yields over 40% after thermal carbonization due to its limited carbon content (commonly 44% at the highest), during hydrothermal carbonization, the carbon amount in the sample increased to approximately 70% depending on the hydrothermal carbonization temperature. This enabled us to obtain high yields after thermal carbonization [4].

**Figure 2:**
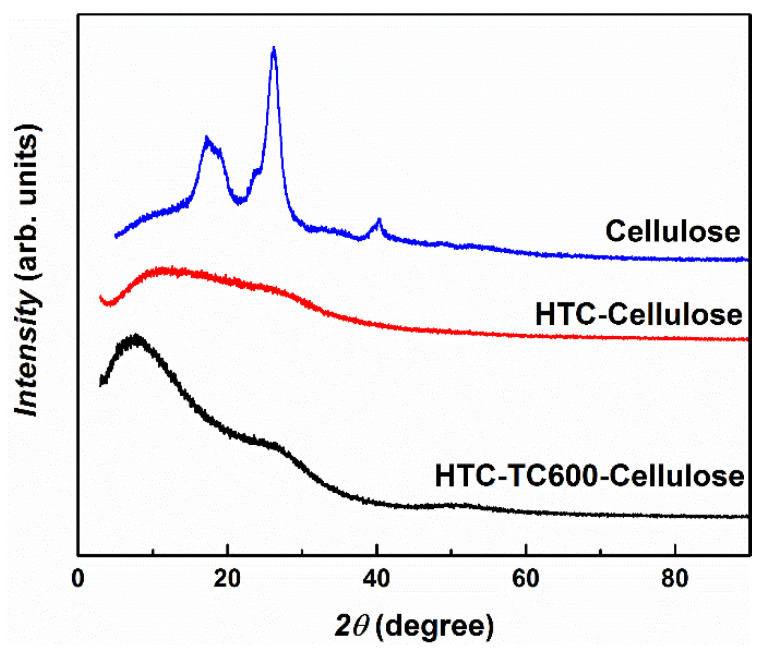
XRD patterns of the prepared particles: cellulose, HTC-Cellulose and HTC-TC600-Cellulose.

XRD analysis was performed to describe the transformation of cellulose during its carbonization. The pristine cellulose exhibited, in its diffractogram, three typical peaks (Figure 2), where the former two represent the crystalline part of the cellulose and the former stands for its longitudinal structure [5]. For the HTC-Cellulose sample, no clear peak was observed since, during hydrothermal carbonization, crystalline structures within the sample were disordered and the amorphous irregular structure was obtained. It has been shown [5] that the critical temperature for hydrothermal carbonization of pristine cellulose is at least 220 °C, and that when the temperature is only slightly lower (210 °C), almost no transformation occurs and the XRD pattern of such material is very close to the XRD pattern of pristine cellulose. The XRD pattern of the HTC-TC600-Cellulose sample exhibited broad diffraction peaks, indicating the amorphous character of the sample. The slightly different shape of the diffractogram may be explained as a consequence of carbonization at the higher temperature that leads to the short-range order of the amorphous structure [40]. Moreover, the additional features of morphology and porosity can be considered to affect the shape of the XRD pattern. The carbonization of the particles further significantly affected the conductivity of the particles. While pristine cellulose and HTC-Cellulose particles were non-conducting with conductivities of 1.09 × 10^−13^ S cm^−1^ and 2.81 × 10^−13^ S cm^−1^, respectively, their carbonized analogue HTC-TC600-Cellulose exhibited a conductivity of 3.48 × 10^−6^ S cm^−1^. This increase in conductivity enables the utilization of HTC-TC600-Cellulose in many applications where conducting polymers are commonly used, and the obtain conductivity is, for example, desirable for ER fluids to exhibit high ER effects.

**Figure 3:**
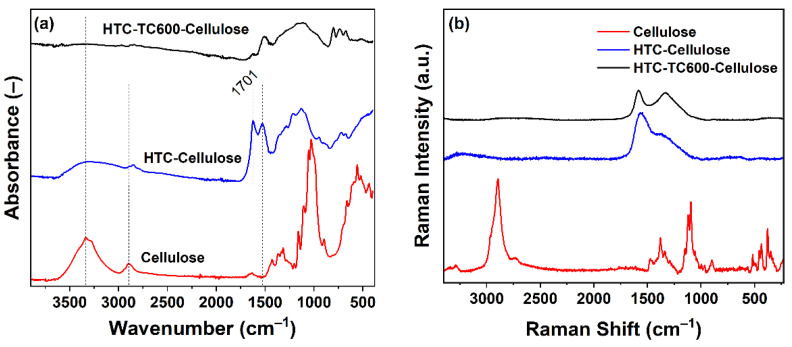
FTIR (**a**) and Raman (**b**) spectra of the cellulose, HTC-Cellulose and HTC-TC600-Cellulose particles.

Fourier transform infrared spectroscopy and Raman spectroscopy were used to analyze the chemical composition changes in the carbonized cellulose when compared to pure cellulose. For pure cellulose particles, a typical spectrum representing cellulose was observed. Namely, the broad spectral band at 3334 cm^−1^ and the spectral band at 2896 cm^−1^ represent hydroxyl group and C–H stretching, respectively, while a dominant band at 1029 cm^−1^ stands for the etheric C–O group present in the ring (Figure 3a) [3]. After the hydrothermal carbonization process (Figure 3a), the intensity of the cellulose wide stretching band attributing to the vibrations of the hydroxyl group at 3320 cm^−1^ decrease, and during the thermal carbonization the peak disappears completely. Furthermore, during hydrothermal carbonization, benzene units are possibly created which is reflected by the bands at 1606 cm^−1^ representing C–C vibration at the aromatic ring [1], a low disordered peak around 1520 cm^−1^, and a peak at 796 cm^−1^ standing for the C–H bond at the aromatic ring [4,5,6]. The presence of the carbonyl group C=O at 1701 cm^−1^ further corresponds well with the wide band at ca 3700–3000 cm^−1^, representing the stretching of the O–H group in the hydroxyl or carboxyl groups, which are possibly formed [4,5] (Figure 3a). The spectrum for HTC-TC600-Cellulose is very disordered, confirming the significant change in the structure during thermal carbonization. A Raman spectrum of cellulose exhibits typical spectral bands (C–O, C–H stretching and bending); however, after carbonization, the Raman spectra of both samples of HTC-Cellulose and HTC-TC600-Cellulose contain bands that can be assigned to carbon-like materials with two characteristic bands representing graphitic (G) and disordered (D) states of carbon (Figure 3b). For HTC-Cellulose, the D peak shoulder can only be found, which can be connected with the incomplete transformation into carbonaceous structures.

**Figure 4:**
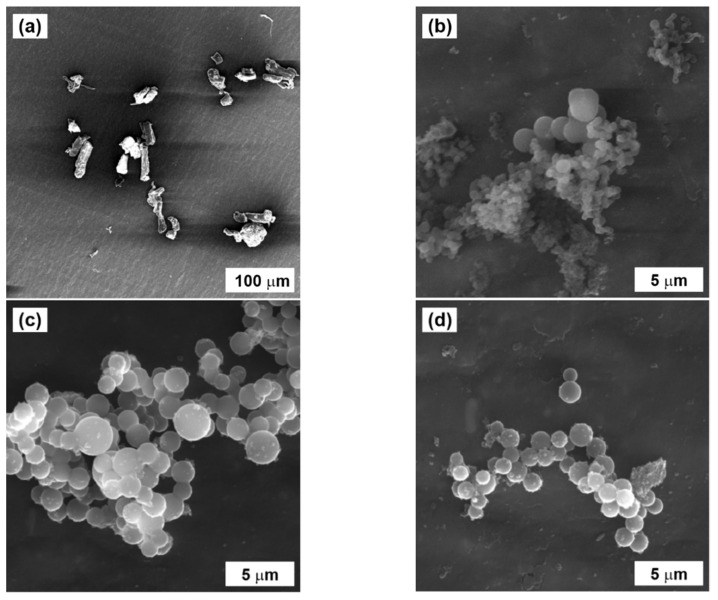
SEM images of (**a**) cellulose, (**b**) HTC-Cellulose, and (**c**,**d**) HTC-TC600-Cellulose particles.

The carbonization process significantly affected particle size and morphology (Figure 4). While particles of pure cellulose were of irregular shape up to 100 µm (Figure 4a), after the hydrothermal process the HTC-Cellulose particles were uniform microspheres sized from hundreds of nanometers up to micrometers (Figure 4b). After thermal carbonization, the particles preserved their spherical shape and size and became more uniform (Figure 4c); nevertheless, it has to be said that the transition does not seem to have been completed, since some irregular complexes were found within the sample (Figure 4d).

**Figure 5:**
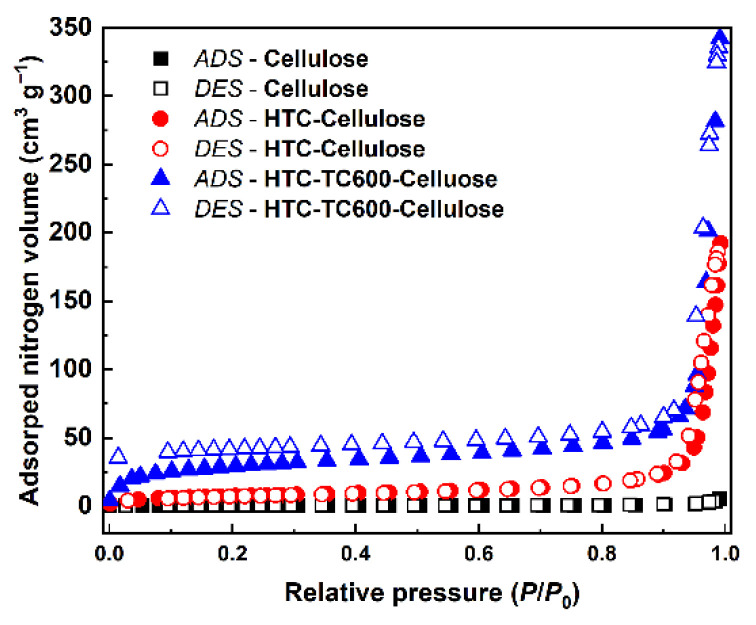
Nitrogen adsorption–desorption isotherms of the tested samples: cellulose, HTC-Cellulose, HTC-TC600-Cellulose. Abbreviations: ADS and DES stands for adsorption and desorption cycle, respectively.

Nitrogen adsorption/desorption isotherms are displayed in Figure 5 and they exhibit type II shape [41] characteristics for microporous carbon-based materials. Based on the results from the BET method, it is shown that the carbonization process increased the surface area, *a_s_*, of the samples by about two orders of magnitude. While pristine cellulose possessed *a*_s_ only 1.22 m^2^ g^−1^, its carbonized analogue HTC-TC600-Cellulose had *a*_s_ 103.7 m^2^ g^−1^, which can significantly increase the ER effect of its ER fluids. Since the adsorption of nitrogen takes place at low pressures and the hysteresis loop was open, it can be assumed that HTC-TC600-Cellulose sample exhibited microporous character [41] (Figure 5). The surface of the HTC-Cellulose sample was 26.6 m^2^ g^−1^, which is only slightly higher than the reference sample.

**Figure 6:**
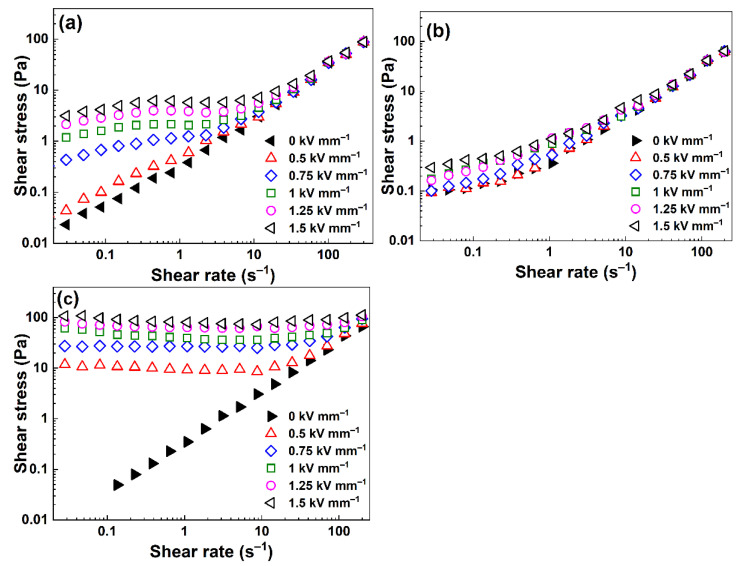
Log–log dependence of shear stress on the shear rate for prepared ER fluids based on (**a**) cellulose, (**b**) HTC-Cellulose and (**c**) HTC-TC600-Cellulose. The concentration of the particles was 5 wt%.

The rheological behavior of the prepared ER fluids at the concentration of 5 wt%, under various external electric field strengths in the range of 0.5–1.5 kV mm^−1^, as well as without it, is presented in Figure 6. In the off-state, the ER fluids displayed almost Newtonian behavior where shear stress increased nearly linearly with the applied shear rate. In the on-state, particles created the internal chain-like structure within the silicone oil leading to an increase in the rheological parameters of the prepared ER fluids, which was most prominent in the ER fluids based on HTC-TC600-Cellulose particles. For the ER fluids based on pristine cellulose or HTC-Cellulose, a slight increase in shear stress at low shear rates reflecting the low ER effect due to poor electrostatic forces among the particles, and thus the low reorganization of the structures, was observed (Figure 6a,b). The ER fluid based on the HTC-TC600-Cellulose particles exhibited a wide plateau of shear stress independent of the shear rate, reflecting a high ER effect due to the increased conductivity of the carbonized particles and the strong electrostatic forces among the particles, which were stronger than the hydrodynamic forces in a broad range of shear rate values.

The fluid containing HTC-Cellulose (Figure 6b) showed a very low ER response due to the low conductivity and disorder of the structure character of cellulose by the hydrothermal carbonization. On the other hand, due to the enhancement of the conductivity after the carbonization process, the ER fluid based on HTC-TC600-Cellulose reached ER effects two orders of magnitude higher than the fluids based on pure cellulose. The value of yield stress, *τ*_y_, expressing the stress which the liquid had to overcome to begin to flow, was more than 100 Pa for the ER fluid based on HTC-TC600-Cellulose at the electric field strength of 1.5 kV mm^−1^ (Figure 6c). The found ER performance is outstanding in comparison to the other published cellulose-based works of research [26]—where such results have not been achieved at such concentrations—and even exceeds most of the so-far-presented results for ER fluids based on other carbonized systems [32,34,42] or state-of-the-art materials [12,43,44]. The increased conductivity of HTC-TC600-Cellulose further influences the leaking current through its ER fluid, determining interactions between the particles. While in the case of ER fluids based on cellulose and HTC-Cellulose, the measured leaking current densities were ~0 µA cm^−2^, in the case of ER fluids based on HTC-TC600-Cellulose, the high leaking current occurred due to the conductivity of the particles, which led to a strong interaction between the particles and the further high ER effects (Table 1). With a higher leaking current, stronger interactions between particles occur and the structure can then withstand higher stress before its distortion.

**Figure 7:**
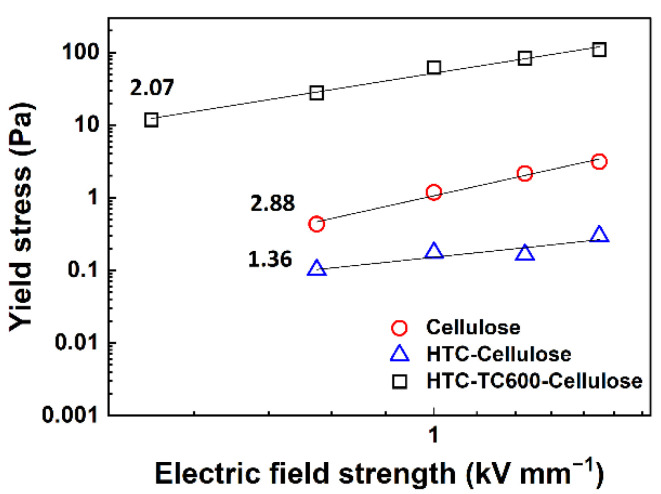
A log–log dependence of yield stress values taken as shear stress at low shear rates on electric field strength for the prepared ER fluids.

The yield stress of ER fluids is proportional to the applied electric field and obeys a power law dependence (Equation (1)) where symbols *τ*_y_, *q*, *E*, and *a* represent yield stress value, internal rigidity of the system, electric field strength, and slope of the curve fitting the data, respectively. In our work, due to simplicity and the low ER effect of ER fluids based on pristine cellulose or HTC-Cellulose, shear stress values at a low shear rate (~0.03 s^−1^) were taken as the values representing yield stress. For the ER fluid based on HTC-TC600-Cellulose, the slope had a value 2.07, which is close to the theoretical polarization model, indicating that the polarization between the particles was the most relevant force directing its ER effect. For the ER fluids based on pristine cellulose or HTC-Cellulose, on the other hand, the values of the slope were 2.88 and 1.36, respectively, which were not possible to connect with either the conduction (slope ~1.5) or polarization models (slope ~2) (Figure 7) [45]. This can be explained by a very weak or nearly absent ER effect; thus, the values of shear stress at low shear rates correspond to chain-like structures that are not fully developed, and which later undergo their reformation and distort the measured values.

(1) $$\tau_{y}\sim q\times E^{a}$$

Dielectric spectroscopy is a suitable tool for the evaluation of ER fluids, as it is closely related to ER activity. After the application of the external electric field, the interfacial polarization occurs in the ER fluids at the particle interface and carrier medium. The polarization rate and the forces generated between the polarized particles are described by a complex permittivity. It has already been proposed [46,47] that the most important quantities connected with ER effects are dielectric relaxation strength, $\Delta \varepsilon^{\prime }$, and relaxation time, $t_{\mathrm{rel}}$, which should be as high and as short as possible, respectively. The Havriliak–Negami model was used to analyze the dielectric behavior of ER fluids according to the following equation (Equation (2)) [48,49,50]:

(2) $$\varepsilon_{\left(\omega \right)}^{*}=\varepsilon_{\infty }^{\prime }+\frac{\left(\varepsilon_{0}^{\prime }-\varepsilon_{\infty }^{\prime }\right)}{{\left(1+{\left(i\omega \cdot t_{\mathrm{rel}}\right)}^{\alpha }\right)}^{\beta }}$$

where $\varepsilon_{\left(\omega \right)}^{*}$ is a complex permittivity, $\varepsilon_{0}^{\prime }$ is a relative permittivity at zero frequency, $\varepsilon_{\infty }^{\prime }$ is a relative permittivity at infinite frequency, ω is angular frequency (2πf), and *α* and *β* are shape parameters both in the range of 0–1, describing the asymmetry of the dielectric function. In the case of the *α* value differing significantly from zero and the *β* value from one, the relaxation is more asymmetrical.

**Figure 8:**
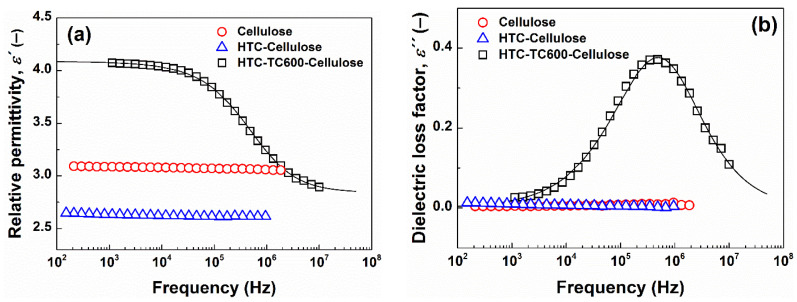
The frequency dependence of relative permittivity, ε′ (**a**) and dielectric loss factor, ε″ (**b**), for 5 wt% silicone oil ER suspensions based on prepared particles. Solid lines represent the Havriliak–Negami model fit.

Frequency dependencies of relative permittivity and the dielectric loss factor of the prepared ER fluids at the concentration of 5 wt% are shown in Figure 8. Among the prepared ER fluids, only the one based on HTC-TC600-Cellulose particles displayed a clear relaxation process in the region of interfacial polarization at higher frequencies, which is desirable for a high ER effect. Its relaxation time was estimated to be 2.52 × 10^−7^ s using the Havriliak–Negami model, with a very high value of dielectric loss factor (~0.4). Hao [51] has proposed that, for a high ER effect, this value should be >0.1 (Figure 8b). The find value ~0.4 is further comparable, and even exceeds values found for most carbonized materials used in electrorheology, showing high ER effects [33,34,36]. For cellulose or HTC-Cellulose-based ER fluids, no dielectric relaxation appears at all in the measured frequency range, which likewise affirms a weak or even non-existent ER effect under the electric field applied. The two-step carbonization of cellulose thus confirmed a proper approach for the preparation of electrically polarizable particles used in ER fluids.

## 3. Materials and Methods

### 3.1. Cellulose Carbonization

**Figure 9:**
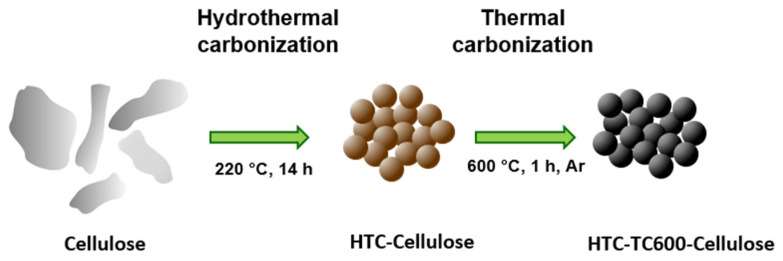
Illustration of the two-step carbonization process of cellulose particles utilizing consecutive hydrothermal and thermal carbonization.

In this study, cellulose particles (fibers, (medium size), moisture content < 10%, Sigma Aldrich; St. Louis, MI, USA) were carbonized in two steps, giving high yields, in order to obtain novel carbonaceous particles from a renewable source (Figure 9). The commercial cellulose was characterized by a content of 67.54% *α*-cellulose and a degree of polymerization (DP = 451) determined using the methods described in the Section 3.2. Particle Characterization. Firstly, hydrothermal carbonization was performed using a teflon autoclave with a total volume of 100 mL. Then, 2 g of cellulose was dispersed in 60 mL of demineralized water and poured into an autoclave. After its fixation in a steel autoclave, the autoclave was put into the oven and heated to 220 °C, which has been demonstrated as the lowest temperature for the transformation of cellulose into microspheres [5]. The hydrothermal carbonization ran for 14 h; afterwards, the autoclave was left to cool down to room temperature in the oven and the yield was thoroughly filtered with demineralized water and ethanol, and subsequently dried at 70 °C in a vacuum. Such particles were labelled as HTC-Cellulose.

Subsequently, the thermal carbonization of HTC-cellulose particles was performed using a tube furnace (Compact 1600c Tube Furnace; MTI Corporation, Richmond, CA, USA). The oven was heated to 600 °C in an argon atmosphere at a heating rate 3.3 K/min and the temperature was kept for 1 h; then, the oven was switched off. The obtained particles were labelled as HTC-TC600-Cellulose. The carbonization temperature of 600 °C was sufficient for the transformation of HTC-Cellulose particles to particles with conductivity high enough for an outstanding ER performance, and mild enough not to short-circuit the measuring instruments [31,34]. A brief scheme of the preparation is captured in Figure 9.

### 3.2. Particle Characterization

The powder XRD diffractograms were recorded on a Rigaku MiniFlex 600 diffractometer (Tokyo, Japan) equipped with a CoKα (λ = 1.7903 Å) X-ray tube (40 kV, 15 mA). Data processing was performed using Rigaku PDXL2 software. The surface area value of the samples was obtained by the physical adsorption of nitrogen gas using a surface area analyzer (Belsorp-mini II, BEL Japan, Inc., Osaka, Japan) using the Brunauer, Emmett and Teller (BET) multipoint method. Samples were outgassed for 18 h at 90 °C prior to the measurements. The prepared particles were further characterized using Fourier transform infrared spectroscopy (FTIR; Nicolet 6700, Thermo Scientific, Waltham, MA, USA) and dispersive Raman microscopy (Nicolet DXR, Thermo Scientific, Waltham, MA, USA). The morphology and size of the particles were observed with a scanning electron microscope (SEM; Vega II LMU, Tescan, Brno, Czech Republic). Thermogravimetric (TGA) analysis was performed in a temperature range of 25–800 °C at a heating rate of 10 K/min under a nitrogen atmosphere using a thermogravimeter (TGA Q500; TA Instruments, New Castle, DE, USA). Conductivity of the compressed powders was measured via a two-point method using an electrometer Keithley (Keithley 6517B, Cleveland, OH, USA).

In the case of pure cellulose particles, their content of *α*-cellulose was determined using the simplified ISO Test Method 692 Pulps-determination of alkali solubility. Briefly, pure cellulose was treated with 17.5% NaOH solution (*w*/*w*) for 1 h at 25 °C. A weight of delignified cellulose (*m*_cellulose_) can be calculated as *m*_cellulose_ = *w*_α_ + *w*_β_ + *w*_γ_, where *w*_α_, *w*_β_ and *w_γ_* stand for weight of α-cellulose, *β*-cellulose and *γ*-cellulose, respectively [52]. Since the latter two are soluble in the alkali mixture, the *α*-cellulose was obtained as a solid fracture using centrifugation and decantation methods after the treatment. The sample was washed several times with 17.5% NaOH solution (*w*/*w*) to remove all the traces, and further washed several times with water and dried overnight at 60 °C. It was found that the used cellulose consisted of 67.54% α-cellulose.

The DP of cellulose was determined using Mark–Houwink equation (Equation (3)), where [η] stands for intrinsic viscosity and *K* and *α* are parameters describing certain polymer–solvent systems.

(3) $$\left[\eta \right]=K\times DP^{\alpha }$$

In this work, a solution consisting of dimethyl sulfoxide/tetrabutylammonium hydroxide/H_2_O in a ratio of 8:1:1 (*w*/*w*) was used as a suitable solvent for cellulose. The solvent dimethyl sulfoxide with purity ≥99.5% was supplied by Sigma Aldrich; St. Louis, MI, USA, as well as tetrabutylammonium hydroxide (~40% in H_2_O). Bu et al. [53] found that the parameters *K* and *α* possess values of 0.24 and 1.21 for the solvent used in Equation (3). The intrinsic viscosity was found to be 391.15 mL/g and, subsequently, the DP of cellulose was calculated to be 451.

### 3.3. Preparation of Electrorheological Fluids and Their Characterization

Electrorheological fluids at the concentration of 5 wt% were prepared by dispersing the cellulose, HTC-Cellulose or HTC-TC600-Cellulose particles within the silicone oil (Lukosiol M200, Chemical Work Kolín, Czech Republic; dynamic viscosity = 194 mPa s at 25 °C). Before the measurements, the prepared fluids were mechanically stirred and then treated using an ultrasonicator (UP400S, Hielscher, Teltow, Germany) at the 30% vibration amplitude after 0.4 cycles for 1 min, which ensured a thorough homogenization. A rotational rheometer Bohlin Gemini (Malvern Instruments, Malvern, UK) with plate–plate geometry (20 mm in diameter with a gap of 0.5 mm) was used to investigate the ER behavior of the prepared ER fluids. The steady shear testing was performed in a controlled shear rate mode in the absence of the external electric field, as well as in the presence of the electric field at the strengths of 0.5–1.5 kV mm^−1^. Before each subsequent measurement, the sample was sheared for 1 min at the shear rate of 20 s^−1^ to destroy any residual structures, and after that the electric field was applied for 1 min to form chain-like structures within a fluid.

Novocontrol Concept 50 (Novocontrol, Montabaur, Germany), an impedance analyzer, was used to investigate the dielectric properties of the prepared ER fluids in the frequency range of 0.003 Hz to 10 MHz. The Havriliak–Negami model was used to analyze the obtained dielectric spectra to obtain the relevant dielectric parameters, as discussed.

## 4. Conclusions

In this study, novel conducting carbonaceous material from the renewable and abundant source of cellulose was prepared by implementing a two-step carbonization process. Firstly, hydrothermal carbonization at 220 °C for 14 h was used, followed by thermal carbonization at 600 °C for 1 h in an argon atmosphere. During thermal carbonization, cellulose is commonly fully combusted even in an inert atmosphere; in our study, the yield of the conducting cellulose-based carbonaceous structure after thermal carbonization was around 45%. The transformation of cellulose into the amorphous carbonaceous microspheres during hydrothermal carbonization was confirmed via XRD analysis, FTIR and Raman spectroscopy. The particles preserved their size and shape after the thermal treatment; however, their surface area increased about two orders of magnitude when compared to pure cellulose and, most importantly, increased from 1.09 × 10^−13^ S cm^−1^ to 3.48 × 10^−6^ S cm^−1^. The particles were further used as a dispersed phase in electrorheological fluids at the concentration of 5 wt%. The suspensions based on two-step carbonized cellulose exhibited a considerably enhanced ER effect when compared to pure cellulose and the values of shear stress were more than 100 Pa at the electric field strength of 1.5 kV mm^−1^, which surpasses the so-far-published results for cellulose particles. The observed electrorheological effects corresponded well to the dielectric properties, which further confirmed the transformation from non-conducting particles to semiconducting carbonized microspheres. The two-step carbonization process of cellulose thus seems to be a novel approach to prepare new carbonaceous materials from renewable sources, with the potential to be used in a variety of industrial applications besides electrorheology, such as adsorbents, catalysts, the stationary phase of liquid chromatography, or electrode materials. The main benefits of the carbonaceous material come from its enhanced conductivity and large surface area, which are desirable in the mentioned applications.

## Figures and Tables

**Table 1:** Leaking current density of the prepared ER fluids.

Electric Field Strength (kV mm^−1^)	Leaking Current Density (µA cm^−2^)		
	Cellulose	HTC-Cellulose	HTC-TC600-CelluLose
0.50	0	0	63.03
0.75	0	0	176.03
1.00	0	0	319.90
1.25	0	0	× *
1.50	0	0	× *

* Leaking currents were over the limit of the measuring device.

## Data Availability

The data presented in this study are available on request from the corresponding author.
